# Protocols for SARS-CoV-2 infection in primary ocular cells and eye organoids

**DOI:** 10.1016/j.xpro.2022.101383

**Published:** 2022-04-22

**Authors:** Anne Zebitz Eriksen, Rasmus Møller, Bar Makovoz, Benjamin R. tenOever, Timothy A. Blenkinsop

**Affiliations:** 1 Department of Cell, Development and Regenerative Biology, Icahn School of Medicine at Mount Sinai, New York, NY 10029, USA; 2 Black Family Stem Cell Institute, Icahn School of Medicine at Mount Sinai, New York, NY 10029, USA; 3 Department of Ophthalmology, Icahn School of Medicine at Mount Sinai, New York, NY 10029, USA; 4 Department of Microbiology, New York University, New York, NY 10016, USA; 5 Department of Health Technology, Technical University of Denmark, 2800 Kgs. Lyngby, Denmark

**Keywords:** Health Sciences, Immunology, Microbiology, Stem Cells, Organoids

## Abstract

Here, we describe a series of protocols detailing the steps for evaluating SARS-CoV-2 infection in models of the human eye. Included are protocols for whole eye organoid differentiation, SARS-CoV-2 infection, and processing organoids for single-cell RNA sequencing. Additional protocols describe how to dissect and culture adult human ocular cells from cadaver donor eyes and how to compare infection of SARS-CoV-2 and the presence of SARS-CoV-2 entry factors using qPCR, immunofluorescence, and plaque assays.

For complete details on the use and execution of this protocol, please refer to Eriksen et al. (2021).

## Highlights

• Identification of SARS-CoV-2 entry factors in cultured donor eye cells
• Revealing cell types vulnerable to SARS-CoV-2 infection in whole eye organoid
• Determination on of infection potential of SARS-CoV-2 in ocular cells
• Protocol enables comparison of infection in donor cells and hESC-derived organoids

## Graphical abstract

**Figure:**
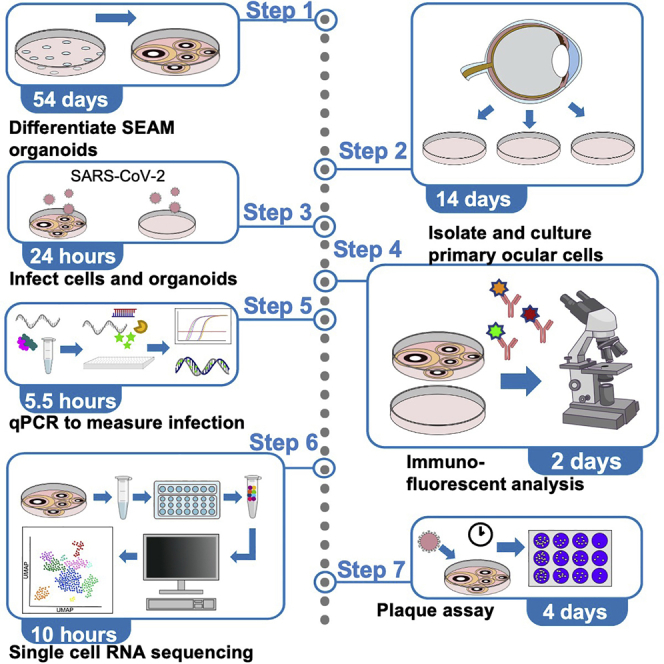

## Before you begin

The protocol below describes how to grow self-formed ectodermal autonomous multizone (SEAM) eye organoids, modified from the original protocol published by Hayashi et al. (Hayashi et al., 2016). Here we describe the differentiation of human embryonic stem cell line H9 into SEAM, used to study infection with SARS-CoV-2 in the eye, as first presented by Eriksen et al. (Eriksen et al., 2021). However, we have also successfully differentiated induced pluripotent stem cell knockout lines and a dCas9-KRAB edited stem cell line using this methodology. If the stem cells are grown on feeder cells, passage the cells into feeder-free conditions for at least two passages prior to initiating SEAM differentiation.

For the adult human cells, cells are freshly isolated from post-mortem donor globes or from cornea donor shells rejected for use as transplants. Tissue should be isolated in preservation solution within 12 h of death and processed for cell culture within 40 h. Shorter time intervals between tissue isolation and processing will result in a higher yield of viable cells. Before obtaining human tissue, make sure your lab follows the institutional guidelines on how to handle and dispose of biohazardous materials and that consent forms from the donor or donor’s family are in place.

Before beginning work with infectious agents such as SARS-CoV-2, be sure the required licenses and safety protocols are in place for your lab. Beware that, different countries and institutions have different guidelines on how to work with infectious agents.

### Institutional permissions

All research was conducted approved for IRB waiver and declared non-human subjects research by the Icahn School of Medicine IRB approving committee which follows the Code of Federal Regulations (CFR), Title 45, Part 46 (45 CFR 46). Anyone replicating protocols must acquire permissions from the relevant institutions in their area.

## Key resources table

**Table:** 

REAGENT or RESOURCE	SOURCE	IDENTIFIER
**Antibodies**		

Mouse anti-SARS-CoV-2 Spike (S)	Center for Therapeutic Antibody Discovery at the Icahn School of Medicine at Mount Sinai	Clone 2B3E5; RRID: AB_431451
Goat Anti-ACE2 antibody	Abcam	Cat# ab15348; Lot GR3333640-8; RRID: AB_301861
Human/Mouse/Rat/Hamster ACE-2 Antibody	R&D systems	Cat# AF933; Lot HOK0320061; RRID: AB_355722
F(ab’)2-Goat anti-Mouse IgG (H+L) Cross-Adsorbed Secondary Antibody, Alexa Fluor 488	Invitrogen	Cat# A11017; Lot 2108802; RRID: AB_143160
F(ab’)2-Goat anti-Rabbit IgG (H+L) Cross-Adsorbed Secondary Antibody, Alexa Fluor 647	Invitrogen	Cat# A21246; Lot 2069609; RRID: AB_1500778
Donkey anti-Goat IgG (H+L) Cross-Adsorbed Secondary Antibody, Alexa Fluor 488	Invitrogen	Cat# A11055; Lot 1463163; RRID: AB_2534102
F(ab’)2-Goat anti-Rabbit IgG (H+L) Cross-Adsorbed Secondary Antibody, Alexa Fluor 546	Invitrogen	Cat# A11071; Lot 1789904; RRID: AB_1500774
F(ab’)2-Goat anti-Mouse IgG (H+L) Cross-Adsorbed Secondary Antibody, Alexa Fluor 647	Invitrogen	Cat# A21237; Lot 1597026; RRID: AB_1500743
Anti-Cytokeratin3/CK-3 antibody [AE5]	Abcam	Cat# ab68260; Lot GR3329790-1; RRID: AB_1140695
Recombinant Anti-Keratin 12/K12 antibody [EPR17882]	Abcam	Cat# ab185627 Lot GR224164-15; RRID: AB_2889825
Cytokeratin 15 Antibody (ST04-05)	Novus	Cat# NBP2-67525; Lot HM-701; RRID: AB_2889827
Anti-STRO1 antibody [7i35]	Abcam	Cat# ab102969; Lot GR8271-1; RRID: AB_10710681
Human Pax6 Antibody	R&D systems	Cat# AF8150; RRID: AB_2827378
Purified anti-Pax-6 Antibody	BioLegend	Cat# PRB-278P; Lot E11EF01096; RRID: AB_291612
Anti-alpha smooth muscle Actin antibody [1A4]	Abcam	Cat# ab7817; Lot GR3257713-4; RRID: AB_262054
Anti-MiTF antibody [D5]	Abcam	Cat# ab3201; Lot GR3356560-1; RRID: AB_303601
Recombinant Anti-Calreticulin antibody [EPR3924]	Abcam	Cat# ab92516 Lot GR3185160-1; RRID: AB_10562796
Purified anti-Tubulin β 3 (TUBB3) antibody	BioLegend	Cat# 801213; RRID: AB_2728521
Sheep anti Human chx 10 (Visual system homeobox 2) (CT)	Exalpha	Cat# X1179P; Lot 13703; RRID: AB_2889828
E-Cadherin (24E10) Rabbit mAb	Cell Signaling Technology	Cat# 3195; Lot 13; RRID: AB_2291471
Recombinant Anti-TMPRSS2 antibody [EPR3862]	Abcam	Cat# ab242384; Lot GR3340286-1; RRID: AB_2889829
SARS-CoV-2 (COVID-19) nucleocapsid antibody	GeneTex	Cat# GTX135357; RRID: AB_2868464
p63 antibody	Santa Cruz	Cat# SC-8431; RRID: AB_628091

**Bacterial and virus strains**		

SARS-CoV-2, isolate USA-WA1/2020	BEI Resources	Cat# NR-52281

**Biological samples**		

Human, male, 60–80 year old wild type eye globes	Eye-Bank for Sight Restoration	N/A
SARS-CoV-2/USA-WA1/2020 isolate	GenBank	Accession no. MN985325.1

**Chemicals, peptides, and recombinant proteins**		

Collagenase	Worthington	Cat# LS004176
Ambion DNase I Solution	Invitrogen	Cat# AM2222
Thiazovivin	Stem Cell Technologies	Cat# 100-0247
Synthemax II	Corning	Cat# 3535
CryoStor CS2 Freeze Media	Sigma-Aldrich	Cat# C3124
TRIzol Reagent	Invitrogen	Cat# 15596026
Matrigel	Corning	Cat# 354230

**Critical commercial assays**		

Chromium Single Cell 3′ Library and Gel Bead Kit v3.0	10× Genomics	Cat# 1000078
Chromium Next GEM Chip G Single Cell Kit	10× Genomics	Cat# 1000127
Chromium Next GEM Single Cell 5′ Library and Gel Bead Kit v1.1	10× Genomics	Cat# 1000167
Chromium Single Cell 5′ Library Construction Kit	10× Genomics	Cat# 1000020
10× Genomics Chromium controller v3.16	10× Genomics	N/A
DNA-free DNA removal kit	Invitrogen	Cat# AM1906
KAPA SYBR FAST qPCR Master Mix Kit Universal	Kapa Biosystems	Cat# KK4601
TruSeq Stranded mRNA Library Prep Kit	Illumina	Cat# 20020594

**Deposited data**		

Mouse Gene Atlas	BioGPS	http://biogps.org/downloads/
Single-cell RNA sequencing of SEAM organoid and SEAM organoid infected with SARS-CoV-2	NCBI GEO	GSE165477
Response to SARS-CoV-2 infection in cornea, limbus and sclera from human donors	NCBI GEO	GSE164073
Additional protocols and sequencing data deposited at Mendeley Data	https://data.mendeley.com/datasets/jgw2mcgb67/1	https://doi.org/10.1101/10.17632/jgw2mcgb67.1

**Experimental models: Cell lines**		

Human: Passage 35–40 H9 ES cells	WiCell	N/A
Adult human RPE cells, Passage 1–3	This paper	N/A
Adult human Cornea cells, Passage 1–3	This paper	N/A
Adult human Limbus cells, Passage 1–3	This paper	N/A
Adult human choroid cells, Passage 1–3	This paper	N/A
Adult human Iris cells, Passage 1–3	This paper	N/A
Adult human Iris cells, Passage 1–3	This paper	N/A
Vero E6	ATCC	Cat# CRL-1586; RRID: CVCL_0574

**Oligonucleotides**		

Primer: Sars-CoV-2 N sgRNA Forward: CTCTTGTAGATCTGTTCTCTAAACGAAC	(Blanco-Melo et al., 2020)	(Blanco-Melo et al., 2020)
Primer: Sars-CoV-2 N sgRNA Reverse: GGTCCACCAAACGTAATGCG	(Blanco-Melo et al., 2020)	(Blanco-Melo et al., 2020)
Primer: *TUBA1A* Forward: GCCTGGACCACAAGTTTGAC	(Blanco-Melo et al., 2020)	(Blanco-Melo et al., 2020)
Primer: *TUBA1A* Reverse: TGAAATTCTGGGAGCATGAC	(Blanco-Melo et al., 2020)	(Blanco-Melo et al., 2020)
Primer: *ACE2* Forward: CGAGTGGCTAATTTGAAACCAAGAA	(Zhang et al., 2020)	(Zhang et al., 2020)
Primer: *ACE2* Reverse: ATTGATACGGCTCCGGGACA	(Zhang et al., 2020)	(Zhang et al., 2020)
Primer: *TMPRSS2* Forward: GTCCCCACTGTCTACGAGGT	(Vidal et al., 2015)	(Vidal et al., 2015)
Primer: *TMPRSS2* Reverse: ATTGATACGGCTCCGGGACA	(Vidal et al., 2015)	(Vidal et al., 2015)

**Software and algorithms**		

Prism 8	GraphPad	http://graphpad.com/
Cell Ranger Single-Cell Software Suite (v3.1)	10× Genomics	https://support.10xgenomics.com/single-cell-gene-expression/software/pipelines/latest/installation
Seurat R package	(Stuart et al., 2019)	(Stuart et al., 2019)
Jensen =TISSUES= text	Jensen Lab	https://tissues.jensenlab.org/Search
bowtie2 R package	(Langmead and Salzberg, 2012)	(Langmead and Salzberg, 2012)
ggplot2 R package	(Wickham et al., 2016)	https://ggplot2.tidyverse.org
R	R Foundation for Statistical Computing,	https://www.R-project.org/
BaseSpace	Illumina	http://basespace.illumina.com/dashboard
DEseq2	(Love et al., 2014)	https://bioconductor.org/packages/release/bioc/html/DESeq2.html
RNA-Seq Alignment App v.2.0.2	Illumina	http://basespace.illumina.com/dashboard

## Materials and equipment

**Table:** mTeSR+ media

Reagent	Final concentration	Amount
mTeSR Plus base media (100-0274, Stem Cell Technologies)	–	39 mL
mTeSR Plus supplement	10%	10 mL
GlutaMAX 100×	1%	0.5 mL
Penicillin-Streptomycin solution (penicillin 10,000 units/mL, streptomycin)	1%	0.5 mL
**Total**	**n/a**	**50 mL**

Filter through 0.2 μm sterile filter, store at 4°C for up to three weeks.

**Table:** SEAM-media

Reagent	Final concentration	Amount
GMEM (11710-035, Gibco)	–	215 mL
Knock-out Serum Replacement	10%	25 mL
GlutaMAX 100×	2 mM	2.5 mL
Non-Essential Amino Acids	0.1 mM	2.5 mL
Na-Pyruvate	1 mM	2.5 mL
Penicillin-Streptomycin solution (penicillin 10,000 units/mL, streptomycin)	1%	2.5 mL
β-Mercaptoethanol stock (1 μL pure β -Mercaptoethanol diluted with 99 μL DMEM:F12)	55 μM	96 μL
**Total**	**n/a**	**250 mL**

Filter through 0.2 μm sterile filter, store at 4°C for up to 1 month.

**Table:** TAB2-media

Reagent	Final concentration	Amount
DMEM:F12 (15-090-CM, Corning)	–	114.75 mL
αMEM (12571-063, Gibco)	–	114.75 mL
Heat inactivated fetal bovine serum (hi-FBS)	2%	5 mL
GlutaMAX 100×	2 mM	2.5 mL
Non-Essential Amino Acids	0.1 mM	2.5 mL
Na-Pyruvate	1 mM	2.5 mL
Penicillin-Streptomycin solution (penicillin 10,000 units/mL, streptomycin)	1%	2.5 mL
Nicotinamide (1 M)	10 mM	2.5 mL
N1 supplement ∗see N1 table	–	2.5 mL
THT stock ∗see THT table (thaw at 50°C to dissolve taurine)	–	0.5 mL
**Total**	**n/a**	**250 mL**

Filter through 0.2 μm sterile filter, store at 4°C for up to 1 month.

**Table:** THT-stock solution (500×)

Reagent	Final concentration	Amount
Taurine	1 M	6.25 g
Hydrocortisone (from 1 mg/mL stock in 70% ethanol)	27.6 μM	0.5 mL
3,3′,5-Triiodo-L-thyronine sodium salt (1 mg/mL in 1 N NaOH)	9.66 nM	0.325 μL
Earle’s Balanced salt solution	–	50 mL
**Total**	**n/a**	**50 mL**

Aliquot, store at −80°C for up to one year.

**Table:** N1 supplement (100×)

Reagent	Final concentration	Amount
Human Transferrin (50 mg/mL in Milli Q water)	0.5 mg/mL	1 mL
Na-Selenite (50 μg/mL in Milli Q water)	0.5 μg/mL	1 mL
Putrescine (160 mg/mL in Milli Q water)	1.6 mg/mL	1 mL
Progesterone (52 μg/mL in 100% Ethanol)	0.52 μg/mL	1 mL
Earle’s Balanced salt solution	–	90 mL
**Total**	**n/a**	**50 mL**

Aliquot, store at −80°C for up to one year.

**Table:** Preservation solution

Reagent	Final concentration	Amount
Potassium Chloride	30 mM	2.24 g
Sodium hydroxide	5 mM	0.2 g
Sodium phosphate monobasic anhydrous	5 mM	0.6 g
Magnesium chloride hexahydrate	0.5 mM	0.1 g
Na-Pyruvate	20 mM	2.2 g
Dextrose	5 mM	1 g
Sorbitol	200 mM	36.4 g
Nicotinamide (1 M)	10 mM	10 mL
Milli-Q water	–	To 1 L
**Total**	**n/a**	**1 L**

Adjust pH to 7.3–7.4 with HCl. Filter through 0.2 μm sterile filter, store at 4°C.

**Table:** Modified DMEM

Reagent	Final concentration	Amount
DMEM (10-013-CV, Corning)	–	470 mL
HI-FBS	2%	10 mL
GlutaMAX 100×	2 mM	5 mL
Non-Essential Amino Acids	0.1 mM	5 mL
Na-Pyruvate	1 mM	5 mL
Penicillin-Streptomycin solution (penicillin 10,000 units/mL, streptomycin)	1%	5 mL
HEPES	1 M	5 mL
**Total**	**n/a**	**500 mL**

Filter through 0.2 μm sterile filter, store at 4°C for up to one month.

**Table:** Thiazovivin working stock solution

Reagent	Final concentration	Amount
Thiazovivin (10 mM in DMSO)	2 μM	0.2 μL
DMEM:F12	–	1 mL
**Total**	**2 μM**	**1 mL**

Store at 4°C for up to 2 months.

**Table:** 2× MEM

Reagent	Final concentration	Amount
10× MEM	–	100 mL
35% BSA solution	0.42%	6 mL
GlutaMAX 100×	2 mM	10 mL
HEPES	1 M	10 mL
5% NaHCO_3_ solution	0.24%	24 mL
Penicillin-Streptomycin solution (penicillin 10,000 units/mL, streptomycin)	1%	10 mL
Milli Q water	–	340 mL
**Total**	**n/a**	**500 mL**

Filter through 0.2 μm sterile filter, store at 4°C for up to one month.

**Table:** Overlay media

Reagent	Final concentration	Amount
HI-FBS	2%	1 mL
2× MEM	–	31 mL
2% Oxoid Agar (LP0028)	–	17.5 mL
**Total**	**n/a**	**49.5 mL**

Filter through 0.2 μm sterile filter, make fresh every time 4°C.

**Table:** Blocking buffer

Reagent	Final concentration	Amount
Normal donkey serum	5%	1.25 mL
Bovine serum albumin	1% (wt/vol)	0.25 g
Triton-X 100	0.3%	75 μL
PBS	–	22.75 mL
**Total**	**n/a**	**25 mL**

Store at 4°C, store for up to 2 weeks.

**Table:** Staining buffer

Reagent	Final concentration	Amount
Normal donkey serum	5%	1.25 mL
Bovine serum albumin	1%	0.25 g
PBS	–	23.75 mL
**Total**	**n/a**	**25 mL**

Store at 4°C, store for up to 2 weeks.

## Step-by-step method details

### Generation of SEAM organoids

**Timing: 31–54 days**

**Figure 1:**
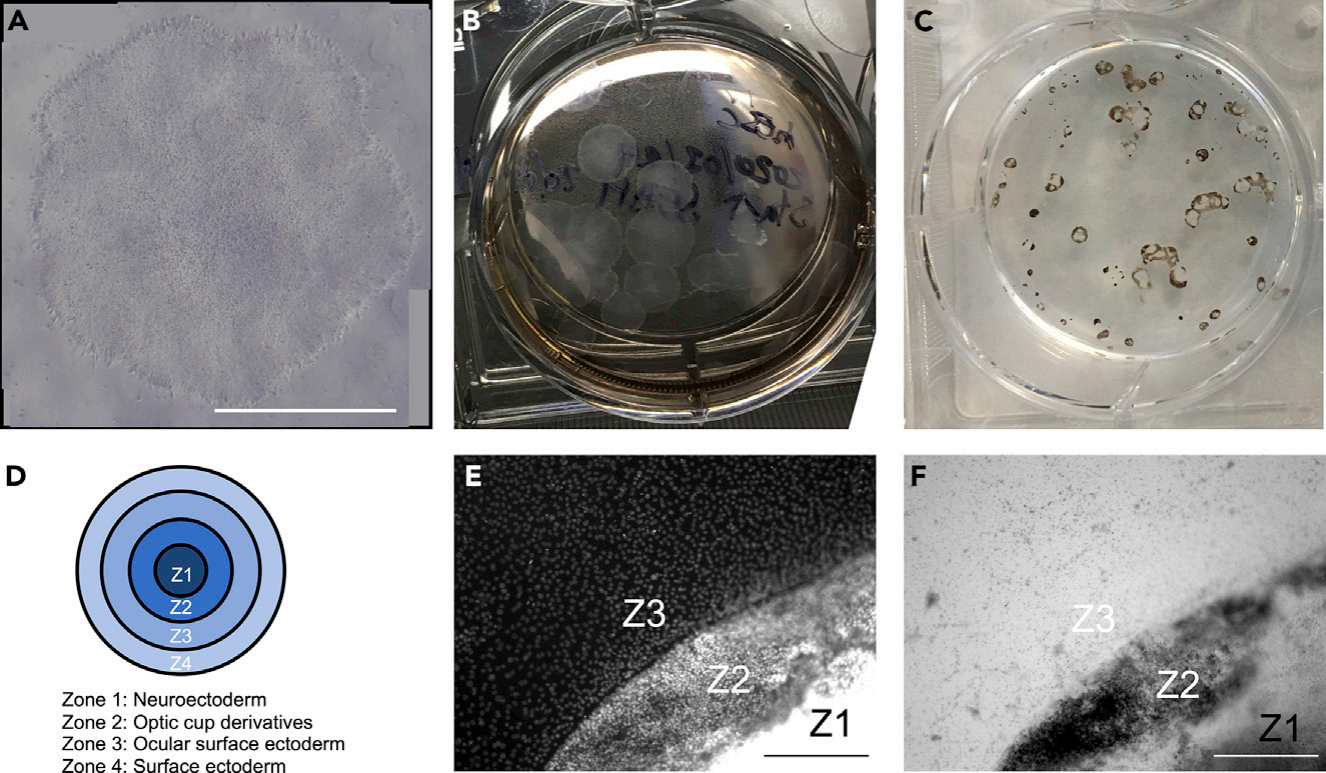
SEAM cultures (A) Human ESC colony before initiating SEAM differentiation, scale bar=1 mm. (B) SEAM colonies 6 days after initiation of differentiation viewed from the bottom of the well, showing a denser middle zone and cells growing out in concentric circles around the middle zone. (C) Mature SEAM colonies at 52 days after initiating differentiation, viewed from the top. The middle zone (zone 1, (Z1)) is dense and appear white, with the pigmented cells of zone 2 (Z2) around. (D) Schematic overview of the four zones of a mature SEAM colony. (E) SEAM colony stained with DAPI showing the different cell density and cell size of zone 1, 2 and 3, scale bar = 500 μm. (F) Bright field image of the images in (E), with visually pigmented cells in zone 2. Scale bar = 500 μm.
3. Initiate SEAM differentiation Day 2.
  a. Warm the SEAM-media to 37°C.
  b. Aspirate half the media in each well and replace it with warm SEAM-media, 1 mL/well.
4. SEAM maturation Day 4–54.
  a. Aspirate total volume of media in each well every other day and replace it with fresh SEAM-media, 2 mL/well.

***Note:*** Early after the initiation of differentiation, colonies should expand and become more circular, with a denser middle and a more see-through outer zone occupied by larger cells, see Figure 1B. After approximately 25 days of differentiation, pigmented cells should become visible in zone 2, see Figure 1C. Further descriptions of the expected SEAM morphology can be found under Expected Outcomes.

1. Passage ESC to seed out colonies for SEAM differentiation, Day -8.
  a. Coat the desired number of wells of a 6-well plate with a 1:80 dilution of Matrigel in DMEM on ice. Use 1–1.5 mL of coating solution in each well and leave the plate to coat for 2 h at 15°C–25°C or at least 1 h in the incubator at 37°C.
    ***Note:*** Keep the Matrigel solution cold until it is distributed in the well plate to avoid gelling. Agitate the plate side to side and front to back to get and even coating.
  b. Prepare 2 mL mTeSR media per well of a 6-well plate to be seeded and add 0.2 μL/mL of Thiazovivin stock solution.
  c. To passage the feeder-free H9 ESCs, begin by warming 0.5 mM EDTA to 37°C in a dry bath.
  d. Wash one well of a 6-well plate containing 80% confluent feeder-free ESCs with 1 mL of EDTA.
  e. Add 1 mL of EDTA and incubate for 2–3 min.
  f. Carefully aspirate the EDTA and add 1 mL of the prepared mTeSR media containing Thiazovivin. Agitate the plate to lift cells into suspension. Carefully wash the well with the cell suspension while maintaining cell aggregates (avoid breaking into single cells).
  g. Take a 10 μL sample from the cell suspension and mix it with 10 μL Trypan Blue, by vigorous trituration to break up cell clusters.
  h. Count the cells using a hemocytometer.
  i. Transfer a volume corresponding to 5,000 cells/well to be seeded to the prepared mTeSR media. Triturate gently to ensure a homogeneous cell density without breaking the colonies down to single cells.
  j. Aspirate the coating solution and seed the cells in the Matrigel-coated wells. Distribute the aggregates by agitating the plate side to side and front to back.
  k. Incubate the cells for eight days at 37°C, changing the media every 1–3 days. Colonies should be 1–2 mm in diameter (see Figure 1A) and be well-separated before initiating differentiation.
    **CRITICAL:** The colonies should be sparse with good space between individual colonies >50 colonies/well to avoid colonies merging before zone 3. See Figure 1B for colonies after 1 week of differentiation.
    ***Note:*** Stem cells should be karyotyped every 10–15 passages, to test for chromosomal duplications, insertions, deletions, and translocations that can impact the differentiation of the cells.

2. Initiate SEAM differentiation, Day 0.
  a. Warm the SEAM-media to 37°C.
  b. Aspirate half the media in each well and replace it with warm SEAM-media, 1 mL/well.

**CRITICAL:** When the culture matures and becomes confluent, extracellular matrix starts forming and some cultures will tend to lift from the plate. Be careful when moving the plate and feeding the cells; avoid agitating the plate to lift cells and collapse the culture (see problem 1 under Trouble shooting).

### Isolation and culture of adult human ocular cells

**Timing: 3–4 weeks**

**Figure 2:**
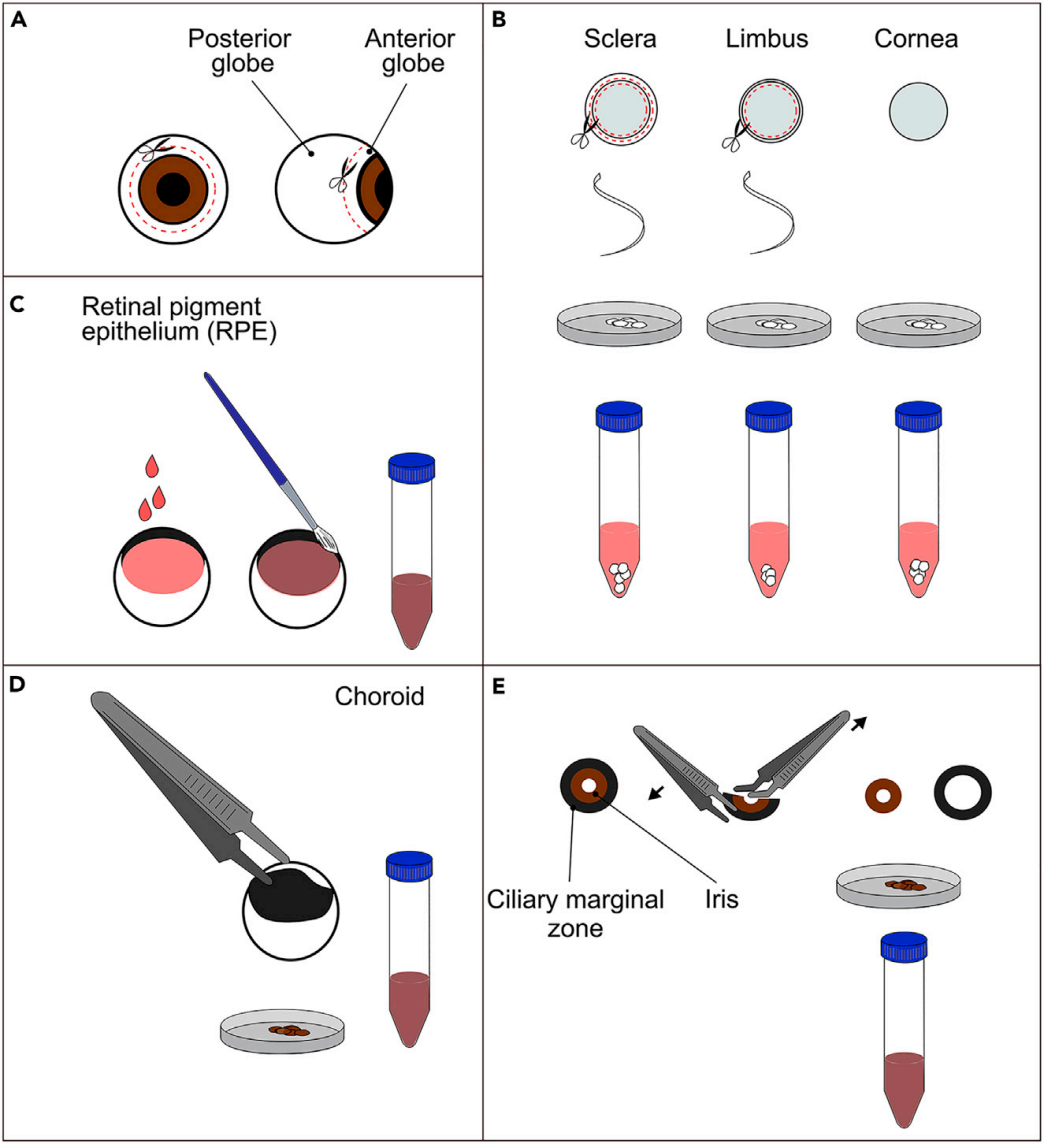
Schematic of ocular dissection Step-by-step illustration from when the shells are received until the initial plating and medium change. (A) Circumferential incision 3–4 mm posterior to the ora serrata. (B) Separating cornea, limbus, sclera and ciliary body/iris using small scissors, cutting individual pieces 1 mm^2^, and placing pieces into separate tubes containing collagenase solution. (C) For the retinal pigment epithelium, remove the retina and add 0.25% trypsin for 45–50 min at 37°C, 5%CO^2^, 20%O^2^. Using a sterile nylon paint brush, brush RPE off from the Bruch’s membrane and collect in a conical tube. (D) Separate the Bruch's membrane and choroid from the scleral wall with forceps. (E) Separate the iris from the ciliary body using forceps, cut into small pieces and place into collagenase solution.
  b. Make a circular cut separating the sclera from the limbus and cornea (Figure 2B Left) and place the scleral tissue in a clean petri dish with sterile PBS to prevent tissue from drying out.
  c. Make a circular cut separating the limbus from the clear tissue of the cornea (Figure 2B middle) and place the limbus and cornea into separate clean petri dishes with sterile PBS to prevent tissue from drying out.
  d. Aspirate the PBS.
  e. Cut the different tissues in to small pieces using a scalpel or a pair of scissors.
  f. Transfer the tissue pieces to the prepared tubes containing collagenase 2 solution. Close The lids loosely to allow gas exchange and incubate at 37°C in the incubator for 2 h, triturating with a p1000 pipette after 1 h of incubation.
  g. Add 300 μL hi-FBS to each tube and triturate with a p1000 pipette to mix.
  h. Pellet the cells by centrifugation at 300 × *g* for 5 min.
  i. Aspirate the supernatant and resuspend cells in 2 mL TAB2 media with 10% FBS and 40 nM thiazovivin. The cell suspension is divided into four wells of a Synthemax-II-SC coated 24-well plate, 500 μL/well.
  j. Incubate cells at 37°C in the incubator 5% CO_2_, 20% O_2_.
11. Isolation of iris.
  a. Cut the ciliary marginal zone from the choroid at the top of the globe, like how the cornea and limbus were separated from the globe in step 10. Transferer the iris and ciliary marginal zone into a clean petri dish with sterile PBS to prevent tissue from drying.
  b. Separate the iris from the ciliary marginal zone by grabbing the iris with an angled pair of tweezers and the ciliary marginal zone with a pair of blunt tweezers, see Figure 2E, and pull the tissues from each other.
  c. Place the iris in a clean petri dish.
  d. Cut the tissue into small pieces using a scalpel or a pair of scissors.
  e. Transfer the tissue pieces into the prepared tube containing collagenase 2 solution. Close The lid loosely to allow gas exchange and incubate at 37°C in the incubator for 2 h, triturating with a p1000 pipette after 1 h of incubation.
  f. Add 300 μL hi-FBS to the tube and triturate with a p1000 pipette to mix.
  g. Pellet the cells by centrifugation at 300 × *g* for 5 min.
  h. Aspirate the supernatant and resuspend cells in 2 mL TAB2 media with 10% FBS and 40 nM thiazovivin. The cell suspension is divided into four wells of a Synthemax-II-SC coated 24-well plate, 500 μL/well.
  i. Incubate cells at 37°C in the incubator 5% CO_2_, 20% O_2_.
12. Isolation of RPE.
  a. Carefully fix the choroid to the sclera by insertion of 3–4 equally spaced 27-gauge needles around the opening of the globe.
  b. Lift out the lens and vitreous. If the retina is not attached to the vitreous, carefully detach the retina from the underlying pigmented cell layer and discard (See problem 2 under troubleshooting if retina does not detach).
  c. Add approximately 3 mL warm trypsin with 40 nM thiazovivin and 3 μL/mL DNase 1 is carefully, filling to globe to roughly 1 mm below the rim of the choroid-RPE (Figure 2C).
  d. Incubate the globe for 45 min.
  e. Carefully detach the RPE cells from the choroid by gentle agitation with a sterilized brush (Figure 2C). Pigmentation in the trypsin should be clearly noticeable when removing the solution from the eye cup and placing it into media.
  f. Transfer the suspension to a centrifuge tube and add 1/10 of the total volume (roughly 300 μL) hi-FBS to inactivate the trypsin.
  g. Pellet the cells by centrifugation at 300 × *g* for 5 min.
  h. Aspirate the supernatant and gently resuspend the cells in 2 mL TAB2 media with 10% FBS and 40 nM thiazovivin. The cell suspension is divided into four wells of a Synthemax-II-SC coated 24-well plate, 500 μL/well.
  i. Incubate cells at 37°C in the incubator.
13. Isolation of choroid.
  a. Gently wash the choroid with sterile PBS to limit RPE contamination.
  b. Peel the choroid from the globe with the blunt tweezers (Figure 2D) and transfer to a clean petri dish.
  c. Cut the tissue in to small pieces using a scalpel or a pair of scissors.
  d. Transfer the tissue pieces to the prepared tube containing collagenase 2 solution. Close the lid loosely to allow gas exchange and incubate at 37°C in the incubator for 2 h, triturating with a p1000 pipette after 1 h of incubation.
  e. Add 300 μL hi-FBS to the tube and triturate with a p1000 pipette to mix.
  f. Pellet the cells by centrifugation at 300 × *g* for 5 min.
  g. Aspirate the supernatant and resuspend cells in 2 mL TAB2 media with 10% FBS and 40 nM thiazovivin. The cell suspension is divided into four wells of a Synthemax-II-SC coated 24-well plate, 500 μL/well.
  h. Incubate cells at 37°C in the incubator 5% CO_2_, 20% O_2_. (See problem 3 under troubleshooting).

***Note:*** The cells should be left to adhere for at least 48 h without disturbing the plate. After this point the media should be exchanged with fresh TAB2 media every 2–4 days.

***Note:*** When expanding the cells, 10% hi-FBS can be added to increase cell proliferation rate. Once confluent, media should return to lower FBS concentration (2%–5%).

***Note:*** When cells are confluent, they can be used for experiments or be further expanded by lifting with trypsin and replating onto Synthemax-II-SC coated plates. All cell types are passaged as described below.

5. Coat four wells of a 24-well plate per donor tissue type to be cultured with Synthemax-II-SC according to the manufacturer guidelines.
6. Prepare one 15 mL centrifuge tube for every tissue type to be processed, except retinal pigment epithelium (RPE), with 3 mL 2.5 mg/mL collagenase II, 0.6 μL thiazovivin (40 nM) and 9 μL DNase 1 (1 μU/mL), and heat to 37°C.
7. Prepare 5 mL trypsin EDTA with 40 nM thiazovivin and 3 μL/mL DNase 1 and heat to 37°C.
8. Prepare 30 mL TAB 2 media with 10% additional hi-FBS and 40 nM thiazovivin and heat to 37°C.
9. If receiving a whole globe, sterilize the globe by submersion in a 30% solution of betadine and then rinsing with sterile PBS twice, before proceeding to dissection under sterile conditions. If receiving a cornea rejected for transplant proceed directly to dissection (step 10b).
10. Isolation of sclera, cornea, and limbus.
  a. Separate the anterior parts of the eye from the posterior part, by making a pars planar circular cut roughly 2 mm posterior to the limbus (Figure 2A).

14. Expansion of cells.
  a. Aspirate the media and wash cells once with Ca^2+^- and Mg^2+^-free PBS.
  b. Add 0.5 mL trypsin to each well to be lifted. Incubate cells with trypsin until they round-up and processes are visibly detaching.
  c. Inactivate the trypsin by adding 0.5 mL 10% hi-FBS in PBS to each well.
  d. Agitate plate to lift cells and transfer cells to a 15 mL centrifuge tube and pellet cells by centrifugation, at 300 × *g* for 5 min.
  e. Aspirate supernatant and resuspend cells in TAB2 with 10% hi-FBS and 40 nM thiazovivin and replate cells 1:4 in Synthemax-II-SC coated plates.
  f. Incubate cells at 37°C in the incubator 5% CO_2_, 20% O_2_.

***Note:*** Cells should be used at low passage numbers (1–4) to avoid morphological and phenotypic changes. Cell identity can be verified with tissue type specific antibody stains, shown in Figure 8. RPE cells should have cobblestone morphology and pigmentation. Scleral cells will be elongated, and cornea and limbus cells will look like fibroblast cells with patches for cobblestone morphology.

### Infection of SEAM cultures and ocular cells with SARS-CoV-2

**Timing: 24 h**

This protocol describes how to inoculate SEAM-cultures with SARS-CoV-2 and maintain the cultures during infection. The same approach is applied when inoculating adult donor cells. When inoculating adult donor cells, serum-reduced SEAM media is swapped for TAB2 media, start at step 16.

15. Prepare 50 mL serum reduced SEAM media.
  a. Mix 47 mL GMEM with 1 mL knockout serum replacement, 0.5 mL GlutaMax, 0.5 mL non-essential amino acids, 0.5 mL penicillin-streptomycin solution, 0.5 mL sodium pyruvate and 19.2 μL β-mercaptoethanol stock (143 mM) to give a final concentration of 55 μM.
  b. Filter the media through a 0.2 μm sterile filter and store at 4°C until use, use within 1 month.

***Note:*** Infections must be performed in media with maximum 2% serum. For infection of adult donor cells, infections should be carried out in TAB2-media. TAB2-media has 2% serum as standard and can be used directly.

16. Warm the serum-reduced SEAM-media to 37°C in a dry bath.
17. In the biosafety cabinet, prepare virus inoculum by diluting SARS-CoV-2 virus stock to 1 M.O.I. in 500 μL SEAM-media per 6-well to be infected.
18. In the biosafety cabinet, aspirate the media from the SEAM culture and replace with the prepared SARS-CoV-2 inoculum. In control wells, replace the media with serum reduced SEAM media without SARS-CoV-2.
19. Place the SEAM-culture plate in the incubator in a humidified atmosphere at 37°C, 5% CO_2,_ 20% O_2_ for one hour and gently rock the plate every ten minutes to allow for even distribution of virus inoculum across the SEAM cultures.
20. After one hour, move the plate back to the biosafety cabinet. Aspirate the inoculum and replace with 2 mL serum-reduced SEAM-media. Incubate the plate at 37°C until the experimental endpoint.
21. Proceed to qPCR, single-cell RNA sequencing (scRNAseq), Plaque assay, or immunohistochemistry protocol.

***Note:*** In all cases except for the plaque assay, quantification of infection was done 24 h post infection. For the plaque assay samples were taken at 12 h, 24 h, 36 h, 48 h and 72 h post infection, see Figure 4A.

**CRITICAL:** All work with SARS-CoV-2 virus must be carried out in a biosafety level 3 facility. When handling the virus, follow all CDC and institutionally approved guidelines. All waste material must be inactivated in an approved disinfectant and autoclaved before discarding.

### Immunohistochemistry

**Timing: 51 h**

This protocol describes how to stain infected cells in 12-well plates, to evaluate infection and expression of the SARS-CoV-2 entry factor ACE2. The protocol can be used for cell type identifying markers and in SEAM cultures as well.

22. Fix cells with 4% PFA.
  a. Aspirate the media from the infected cells and control cells.
  b. Wash cells 3 times with 1 mL/well PBS.
  c. Remove PBS and add 1 mL of ice cold 4% PFA in PBS to each well. Seal the plate with parafilm and fix for a sufficient amount of time, following a protocol approved by your institution to ensure that the virus is completely inactivated, for our institution this is 24 h, at 4°C.
23. Aspirate the PFA solution and wash the cells 3 times with 1 mL/well PBS.
24. Stain the cells with mouse anti-SARS-CoV-2-S and rabbit anti-ACE2.
  a. Block for unspecific antibody binding.
    i. Prepare blocking buffer, (See materials and equipment section).
    ii. Aspirate PBS and add 0.5 mL blocking buffer to each well. Leave cells to block for 1 h on a bench rocker at 15°C–25°C.
  b. Add primary antibodies.
    i. Prepare staining buffer.
    ii. Prepare dilutions of primary antibodies in staining buffer, 0.25 mL/well; mouse anti-SARS-CoV-2 (1:100), rabbit anti-ACE2 (1:100).
    iii. Aspirate blocking buffer and incubate cells with primary antibodies on bench rocker at 4°C 12–18 h.
  c. Wash and add secondary antibodies.
    i. Aspirate the buffer and wash the cell by adding 0.25 mL/well fresh staining buffer and incubate on bench rocker for 5 min at 15°C–25°C. Repeat for a total of 3 washes.
    ii. Prepare secondary antibodies diluted in staining buffer, 0.25 mL/well, goat-anti-mouse Alexa Fluor-488 (1.1000) and goat-anti-rabbit Alexa Fluor-647 (1:500), lastly add the nuclear stain, DAPI (1:1000).
    iii. After the last wash, aspirate the buffer and add 0.25 mL staining buffer with secondary antibody stains to each well. Incubate for 1 h on bench rocker at 15°C–25°C. Protect the plate from light to avoid bleaching of the fluorophores from this step onwards.
    iv. Aspirate the buffer and wash 3 × 5 min with PBS.
  d. After the final wash step, add PBS to cover cells. If proceeding directly to imaging, add minimal volume of PBS to cover cells for improved contrast in bright field images.

***Optional:*** For better, high-resolution imaging, SEAM cultures, and cells intended for immunohistochemical investigation, can be grown on glass bottom plates or cover slips.

**Pause point:** Stained cells can be stored in sealed plates at 4°C for up to two months. When storing cells, add PBS to prevent the plate from drying and 0.1% Na-azide to prevent bacterial growth.

### Measuring infection in by qPCR

**Timing: 5.5 h**

This protocol describes how to quantify expression levels of SARS-CoV-2 entry factors *ACE2* and *TMPRSS2* and SARS-CoV-2 infection in adult donor ocular cells. This analysis is performed on fully confluent cells grown in 24-well plates and infected with 1 M.O.I. of SARS-CoV-2 24 h prior to starting this step of the protocol. The same protocol can be used for quantification of infection in SEAM cultures, adjusting the volumes to accommodate the larger well format in the lysis step.

25. RNA extraction using TRIzol, adapted from Pub. No. 0001271 B.0.
  a. Aspirate cell media and wash 3 times with 0.5 mL PBS.
  b. Lyse cells by adding 500 μL TRIzol reagent (Invitrogen) to each well and triturate the cells vigorously to lyse all cells before transferring the cell lysate to a clean 1.5 mL tube.
  c. Incubate for 5 min at 15°C–25°C.
    **Pause point:** Samples can be stored at −80°C for at least 1 year.
  d. Add 100 μL chloroform to each tube, cap the tube thoroughly, and vortex the samples for 30 s.
  e. Incubate samples for 5 min at 15°C–25°C.
  f. Precipitate the organic phase by centrifugating at >12,000 × *g* for 15 min.
  g. Transfer the aqueous phase (the top phase) to a new tube. Be careful to not contaminate the aqueous phase with material from the organic phase.
  h. Add 5 μL RNase-free glycogen to the sample and mix by inverting ten times.
  i. Add 250 μL isopropanol, mix by inverting ten times and incubate for 10 min on ice.
  j. Precipitate the RNA by centrifugating at > 12,000 × *g* for 10 min at 4°C.
  k. Discard the supernatant.
  l. Dissolve the pellet in 75% ethanol and re-pellet by centrifugating 12,000 × *g* for 5 min at 4°C.
  m. Discard the supernatant and leave the tube open to dry the pellet.
  n. Resuspend the dry pellet in 20 μL RNase-free water.

26. Remove DNA from sample with DNA-free DNA removal kit (Invitrogen) following the protocol of the manufacturer (Cat. No. AM1906 (ThermoFisher)).
  a. Add 2 μL 10× DNase Buffer and 1 μL DNase I to each sample.
  b. Incubate the samples at 37°C for 30 min.
  c. Add 2.5 μL DNase Inactivating reagent to each sample and incubate for 2 min mixing every 30 s.
  d. Spin for 1.5 min at 12,000 × *g* and transfer supernatant to a clean tube.
  e. Determine RNA concentration on NanoDrop (Thermo Scientific) or by using a bioanalyzer (Agilent).
27. Reverse transcribe RNA to cDNA using Superscript II. (Cat. No: 18064-022 (Invitrogen)).
  a. Based on the results from the NanoDrop find the samples with the lowest concentration of RNA. From this sample calculate the amount of RNA in 6 μL of the sample. Based on this amount calculate the volume of RNA to take from additional samples so the amount of RNA is equivalent.
  b. Take the desired volumes of each RNA sample and transfer to clean PCR tubes.
  c. Adjust the volume of all samples to 12 μL with RNase-free water.
  d. Add 1 μL oligo d(T)_12-18_ to each sample.
  e. Add 1 μL dNTP Mix to each sample.
  f. Incubate the samples for 5 min at 65°C, before snap cooling on ice.
  g. Spin the samples shortly to collect evaporation from the cap.
  h. Prepare a Master Mix of 4 μL 5× first stand buffer, 2 μL 0.1 M DTT and 1 μL RNaseOUT per sample.
  i. Add 6 μL Master Mix to each sample and mix gently by triturating.
  j. Incubate 2 min at 15°C–25°C.
  k. Add 1 μL SuperScript II to each sample and incubate samples at 42°C for 50 min.
  l. Stop the reaction by incubating at 70°C for 15 min.

**Pause point:** cDNA samples are stable and can be stored at −20°C for up to 1 year.

**Figure 3:**
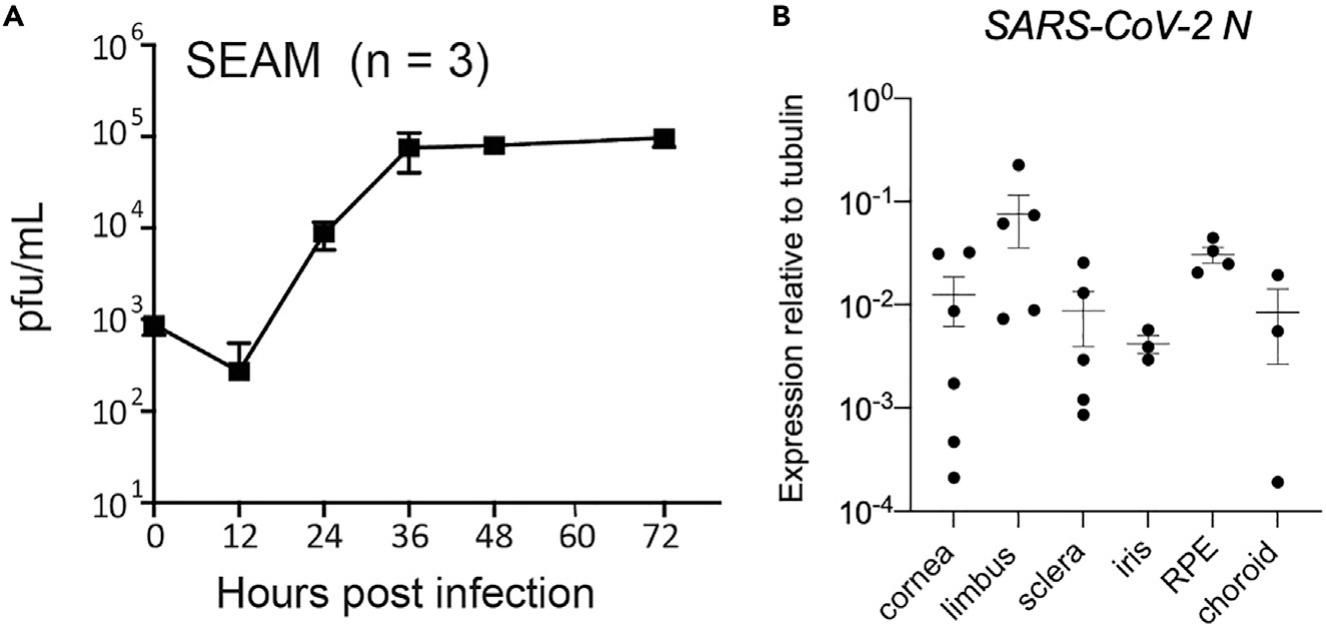
SARS-CoV-2 infection in SEAM and ocular cells (A) Virus growth in SEAM cultures shown as plaque-forming units per milliliter of supernatant at the time points indicated. Error bars show SD (n = 3). (B) qRT-PCR for SARS-CoV-2 subgenomic N transcript on total RNA extracted from ocular tissues infected with SARS-CoV-2. Error bars = SEM. Originally published in (Eriksen et al., 2021).

28. qPCR using KAPA SYBR green fast qPCR Master Mix kit, protocol adapted from manufacturer’s guidelines: (Cat. No. KK4601 (Merck)). Expected outcome for quantification of subgenomic N transcript normalized to tubulin from different ocular cells can be seen in Figure 3B.
  a. Prepare primer mixes for each of the genes of interest (SARS-CoV-2, ACE-2, TMPRSS2 and bTubulin) by mixing forward and reverse primers 1:1. For primer sequences see key resources table.
  b. In a 384-well plate, pipette out 1 μL of the cDNA samples in triplicate so that there are 3 replicates for every sample and primer set.
  c. Add 1 μL of the appropriate primer mix in each well.
  d. Add 8 μL PCR grade water to each sample.
  e. Add 10 μL KAPA SYBR fast qPCR 2× into each sample and mix gently by pipetting up and down.
  f. Seal the plate with a clean adhesive strip and spin the plate for 2 min at 1,000 × *g*.
  g. Perform PCR reaction as described in the table below.

**Table:** 

PCR cycling conditions			
Steps	Temperature	Time	Cycles
Initial Denaturation	98°C	30 s	1
Denaturation	98°C	10 s	42 cycles
Annealing	55°C	20 s	
Extension	72°C	2 min	
Final extension	72°C	1 min	1
Hold	4°C	forever	

### Single-cell RNA sequencing

**Timing: 10 h +**

This step describes how to dissociate SEAM cultures into a single cell suspension ready to be processed for single-cell RNA sequencing.

29. Dissociate SEAM culture to form a single cell suspension.
  a. Prepare collagenase II stock solution. Weigh out 25 mg of collagenase II in a 15 mL centrifuge tube. Dissolve the enzyme in 5 mL Ca^2+^- and Mg^2+^-free PBS and filter the solution through a 0.2 μm filter in sterile conditions.
    ***Note:*** The collagenase stock solution should be stored at 4°C and used within 1 month.
  b. Prepare digestion buffer. For every 1 well of a 6-well plate to be dissociated, mix 1 mL of the collagenase II stock solution, 993.6 μL Ca^2+^- and Mg^2+^-free PBS 0.4 μL thiazovivin (to give a final concentration of 40 nM) and 6 μL DNase I.
  c. Prepare preservation media. Mix 5 mL DMEM:F12 with 1 μL thiazovivin (to give a final concentration of 40 nM), 0.2 μL BSA (from 25% stock), and 15 μL DNase I.
  d. Aspirate the media from the well and wash the cells with Ca^2+^- and Mg^2+^-free PBS.
  e. Add 2 mL digestion buffer to the well and incubate the place at 37°C for 30 min.
  f. Triturate the cells using a p1000 pipette, and incubate for an additional 30 min at 37°C.
    ***Optional:*** If cells are not dissociating and large clumps are visible, add 0.5 mL fresh digestion buffer and incubate for an additional 30 min.
  g. Triturate using a p1000 pipette the cell to break clumps down to single cells, transfer the cell suspension to a 15 mL centrifuge tube, and pellet the cells by spinning at 300 × *g* for 5 min.
  h. Aspirate the supernatant and resuspend the pellet in 2 mL preservation media and filter the suspension through a 40 μm cell strainer.
  i. Count the cells and viability using a hemocytometer and Trypan Blue.
    **CRITICAL:** Only if there is >10,000 cells and the viability is >80% continue to single-cell library preparation.
  j. Preparation of single-cell library according to the manufacturer’s instructions (Cat. No: CG000331 Rev C, 10 Genomics). Data analysis can be performed in R using the Seurat package (Stuart et al., 2019). A code workflow is available on NCBI GEO GSE165477.
    **CRITICAL:** The subgenomic RNAs of SARS-CoV-2 all have unique 5′ ends but share the same 3′ end. Hence, it is crucial to use a library prep kit that is based on 5′ capture of transcripts such as Chromium Next GEM Single Cell 5′ Kit v2 (10×, cat no. RN-10000265) in order to be able to distinguish between viral gene transcripts (Cohen et al., 2021).
    ***Note:*** The 10× protocol provides several possible pause points so that library preparation can be split up over several days if needed.

### Plaque assay

**Timing: 4 days**

Plaque assays are performed to show that a viral infection is productive and that there is true replication of the virus in the infected cells. Here we describe how to perform a plaque assay on infected SEAM cultures to show productive infection in the eye organoids; however, plaque assays can be carried out on any cell type grown and infected *in vitro*. The plaque assay protocol is also used to titer the concentration of infectious virus used for infection experiments.

**Figure 4:**
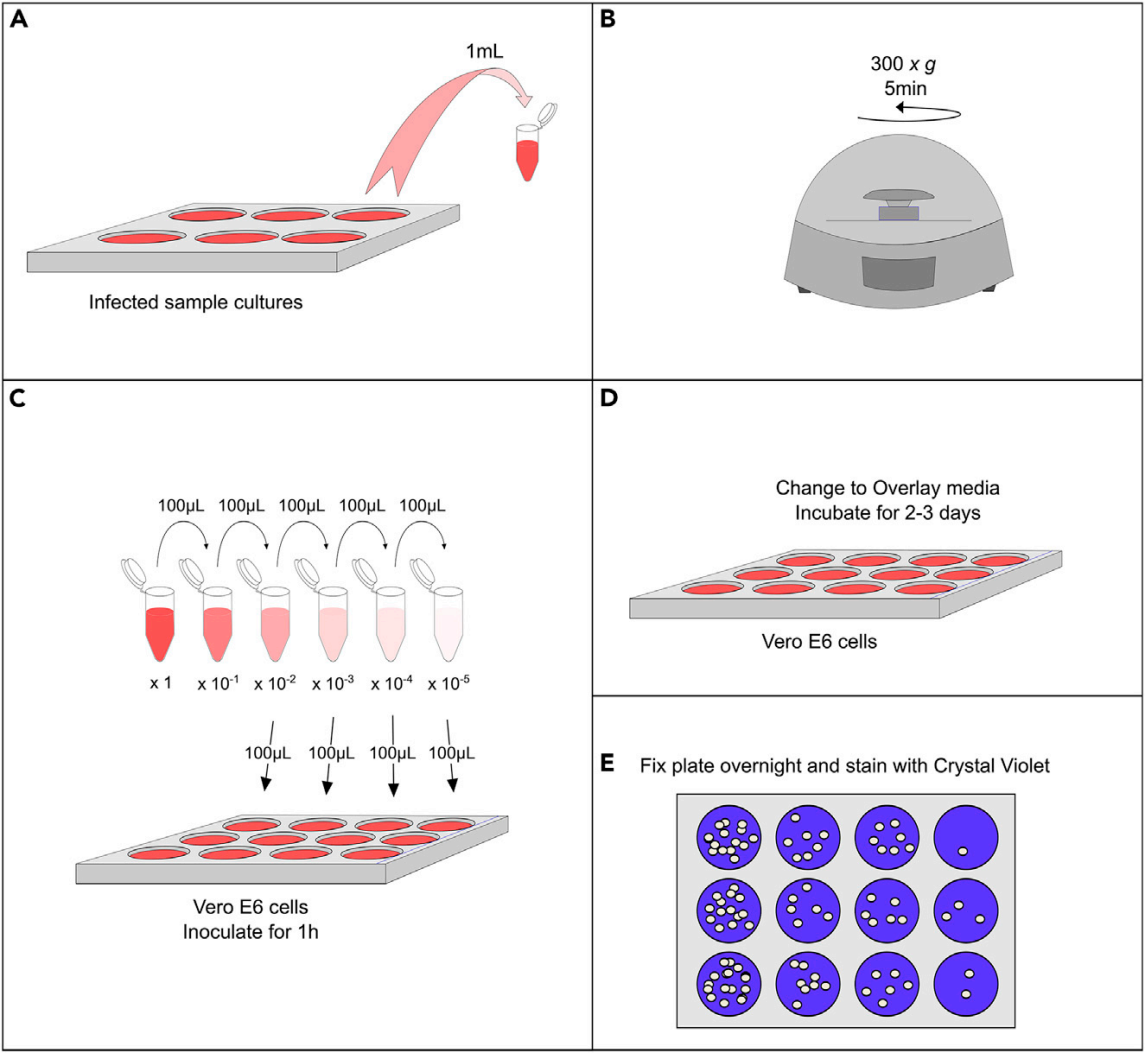
Schematic of the workflow of a plaque assay (A) A sample of the cell media from infected cells is collected. Repeat the sample collection at different time points. (B) Spin sample to pellet dead cells and debris. (C) Make dilution series of media sample and add samples to Vero E6 cells in culture. (D) Add overlay media and incubate for 2–3 days. (E) Fix plate and stain with crystal violet, infected colonies will be visible as white dots.
32. Spin the samples for 5 min at 300 × *g* to pellet any cells and transfer the supernatant to a clean 1.5 mL tube, Figure 4B.
33. Make a logarithmic dilution series of the conditioned media samples.
  a. To make a 10^-1^X sample, take 100 μL conditioned media and dilute it with 900 μL Modified DMEM, Figure 4C.
  b. To make a 10^-2^X sample, take 100 μL of the 10^-1^X sample and dilute it with 900 μL Modified DMEM.
  c. Continue as described above until a dilution of 10^-6^.
34. Inoculate the Vero E6 using conditioned media samples.
  a. Aspirate the media and replace with 900 μL modified DMEM.
  b. Add 100 μL diluted infected sample media in each well.
  c. Allow virus adsorption by incubating for 1 h at 37°C 5% CO_2_, agitating the plate every 10 min.
35. Add solid overlay.
  a. Aspirate the inoculation media.
  b. Add 1 mL/well overlay media, Figure 4D.
  c. Incubate for 48–60 h at 37°C 5% CO_2_.

***Note:*** Make overlay media with oxoid agar fresh every time.

30. Seed Vero E6 cells at 400,000 cells/well in a 12 well plate and incubate cells at 37°C 5% CO_2_ 12–18 h.
31. From the infected SEAM cultures, take 1 mL of the used media from each well and transfer to a clean 1.5 mL tube, Figure 4A.

36. Fix the plates by adding 1 mL/well 4% PFA and incubate for a sufficient amount of time to ensure complete inactivation of infectious virus approved by your institution.
37. Remove the fixative and stain each well by adding 1 mL/well crystal violet solution in 20% ethanol and incubate for 15 min at 15°C–25°C.
38. Aspirate the staining solution and determine viral titers (PFU/mL) by counting white colonies of dead cells in each sample, Figure 4E (see problem 5 under troubleshooting).

## Expected outcomes

Following the differentiation protocol for SEAM organoids adapted from the original publication from Hayashi et al. (Hayashi et al., 2016), we obtain successful SEAM organoids with nice zone formation (see Figures 1C–1F) in 90% of wells were differentiations are initiated.

**Figure 5:**
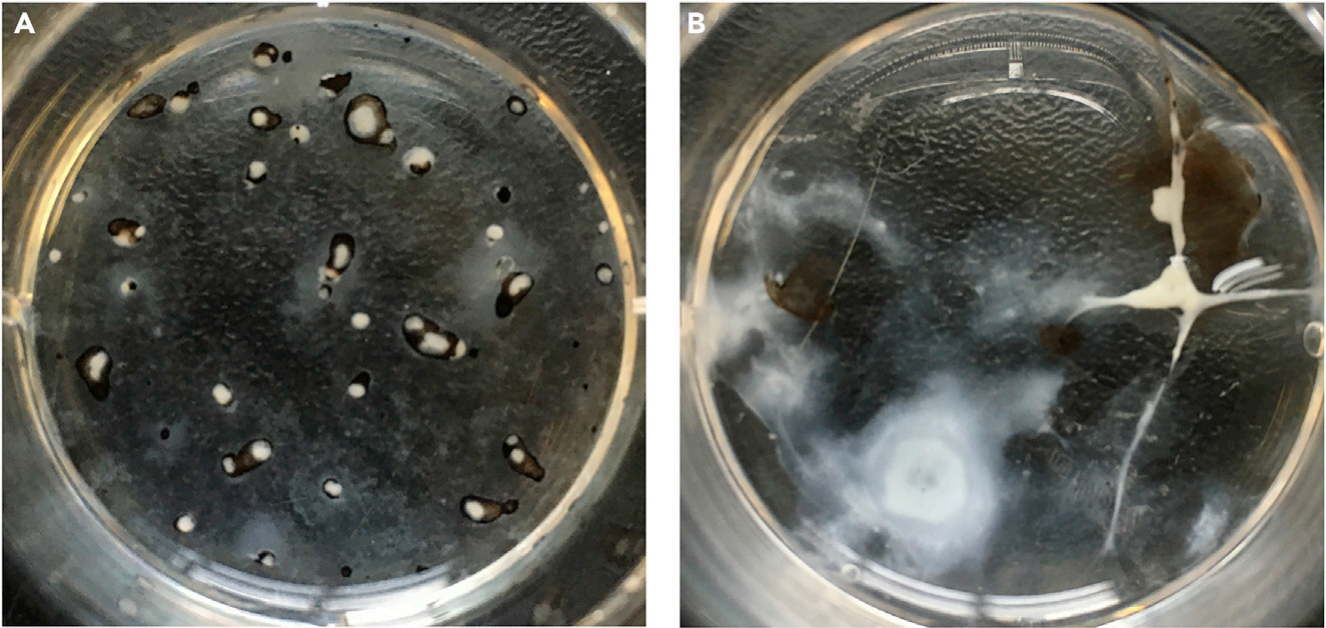
SEAM cultures (A) Successful formation of SEAM organoids, note the white round middle zones (zone 1) surrounded by a strongly pigmented round zone (zone 2). (B) Unsuccessful formation of SEAM cultures with no clear zone formation despite presence of pigmented cells.

**Figure 6:**
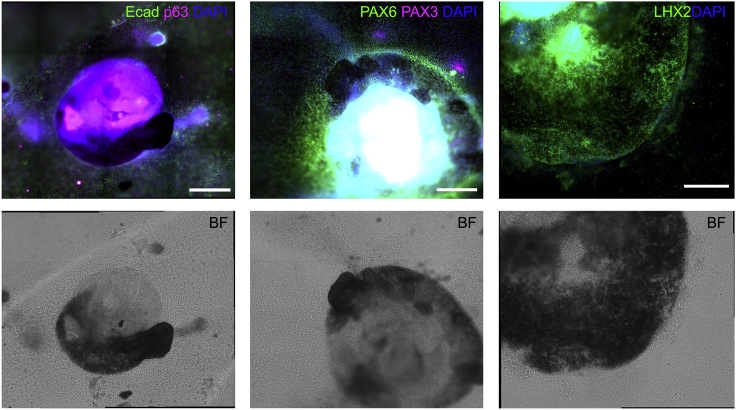
Protein expression pattern in SEAM cultures Zone 3 consisting of ocular surface ectoderm positive for E-cadherin (Ecad) and p63. The eye field transcription factor PAX6 is expressed both in zone 2 and 3, and PAX3 can be found sporadically in zone 3. Another eye field transcription factor LHX2 is strongly expressed by the neuroectoderm derived cells found in zone 2. Scale bar = 300 μm.

**Figure 7:**
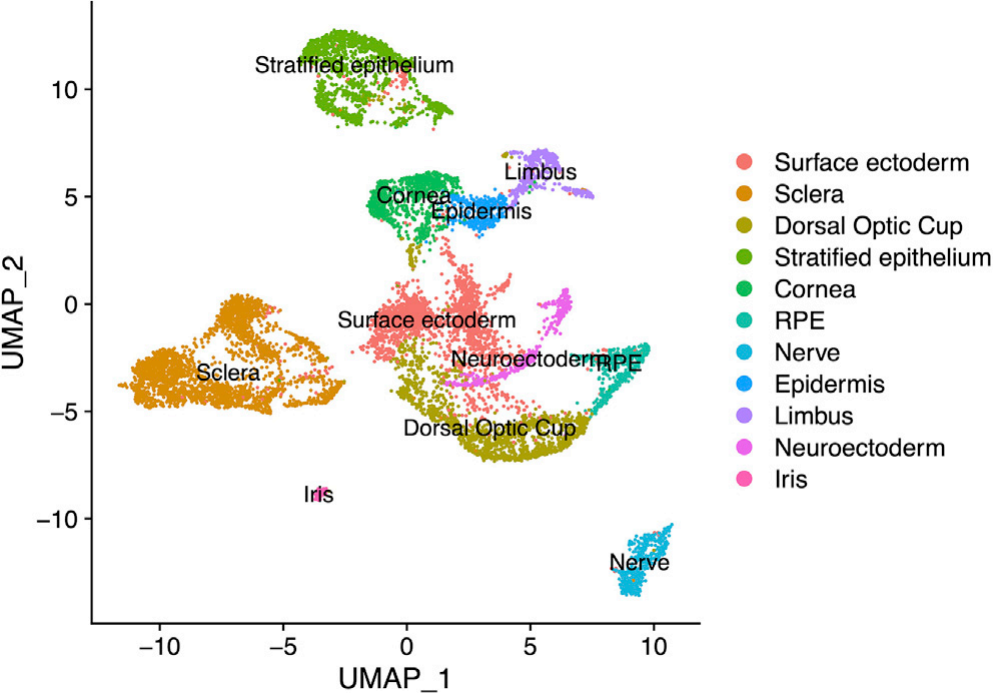
▪▪▪Single cell RNA-sequencing of SEAM organoids Annotated UMAP of two integrated scRNA-sequencing data sets from one healthy SEAM culture and one SEAM culture infected with SARS-CoV-2, as presented in (Eriksen et al., 2021).

Mature SEAM cultures will have obvious zone formation, with round centers (zone 1) surrounded by a clearly pigmented zone (zone 2) (Figure 5). The Zones will have distinct protein expression patterns with markers related to cell fate specification (Figure 6). Single cell RNA sequencing analysis of mature SEAM cultures result in a UMAPs where most of the major cell types of the eye can be recognized in the separated clusters, see Figure 7.

Infection yields of SEAM cultures using SARS-CoV2 virus was 100% (5 out of 5), and no visible signs of severe cell death in the organoid cultures were observed for at least until 72 h post infection.

**Figure 8:**
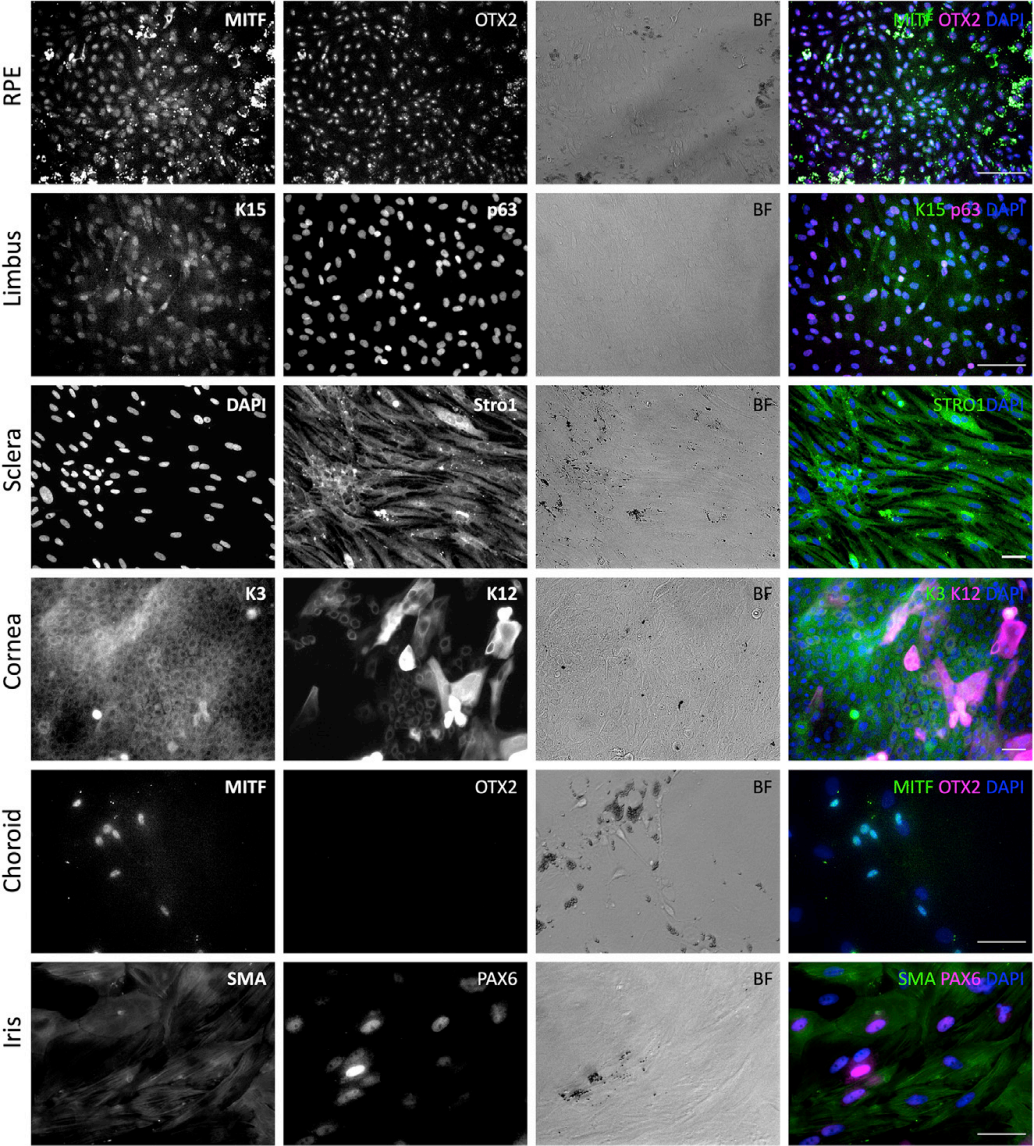
Primary cells isolated from adult donor globes and relevant cell type marker proteins From the top: Retinal pigment epithelium (RPE) cells stained for melanocyte inducing transcription factor (MITF) and Orthodenticle Homeobox 2 (OTX2). Limbus cells stained for Cytokeratin 15 (K15) and tumor protein 63 (p63). Scleral cells stained with mesenchymal precursor marker STRO-1. Corneal cells stained with corneal specific cytokeratin 3 and 12 (K3 and K12). Choroid cells positive for MITF and negative for OTX2. Iris cells positive for smooth muscle actin (αSMA) and paired homeobox 6 (PAX6). Scale bar = 100 μm.

Adult ocular cells isolated from donor eye globes consistently form confluent monolayers, however proliferation potential is highly dependent on the quality, and time between death to isolation. The resulting cell types will have different morphologies and have different expression of identifying specific cell type markers (See Figure 8).

## Limitations

The protocols described here outline how to grow both primary adult ocular cells isolated from donor eye globes, and SEAM eye organoids that simultaneously represent all major cell types of the eye and evaluate infections and infectability with SARS-CoV-2 in these *in vitro* models. The protocol enables the side-by-side comparison of infectability between adult primary cells while also validating results from a stem cell-derived whole eye organoid model. Using these models in tandem enables greater confidence in overlapping results. Moreover, the stem cell-derived SEAM culture offers an examination of the interrelationships between developing cell fates resembling *in vivo* dynamics. However, an *in vitro* model will never be able to truly represent the complexity of the human eye. It should be noted that the SEAM cultures consist of maturing cells, and cells are still not fully mature at day 55. For instance, we do not see high expression of the corneal specific cytokeratins, keratin 3 and keratin 12 in the SEAM corneal cells even though other cornea markers are present. Further maturation of corneal cells can be obtained by following the original protocol by Hayashi et al. (Hayashi et al., 2016), however this method requires the removal of zone 1 and 2 and limits the diversity of cell types that can be tested simultaneously. Another option could be using spheroid organoid culture systems that show better maturation of corneal cells. This approach, however, increases the complexity of the method and might also limit the variety of cell types present in the organoids.

Additionally, worth mentioning is this study only used the H9 embryonic stem cell line. Therefore, infections of SEAM were only performed on that one genetic background. We have successfully differentiated SEAM organoids from other stem cell lines, including human induced pluripotent stem cells (iPSCs). While the infection has not been confirmed in iPSC derived SEAMs, successful differentiation into SEAM organoids and demonstration of cellular diversity has been confirmed.

When performing an infection experiment, it is important to consider the accessibility of the different cell types in the model compared to the native organ. To that effect, it is important to note that the SEAM organoid grows primarily in 2 dimensions. Therefore, viral accessibility is equal between all cell types, and a more accurate representation of a cell’s innate ability to become infected is revealed. However, context should be kept in mind and though a cell type can become infected, whether that cell type would become infected in a real-life scenario, is an open question. Additional factors including exposure time, viral titer and viral accessibility must be considered. For example, the ocular surface (cornea, limbus and sclera) is directly exposed to infectious agents in the air while the retina, RPE and iris are protected.

Finally, an important aspect not recapitulated by the SEAM culture is the tear film turn over that acts as a first line defense in the eye, although we do see some expression of mucosal proteins in the limbus cells in the SEAM.

## Troubleshooting

### Problem 1

Generation of SEAM Organoids, step 4.

**Figure 9:**
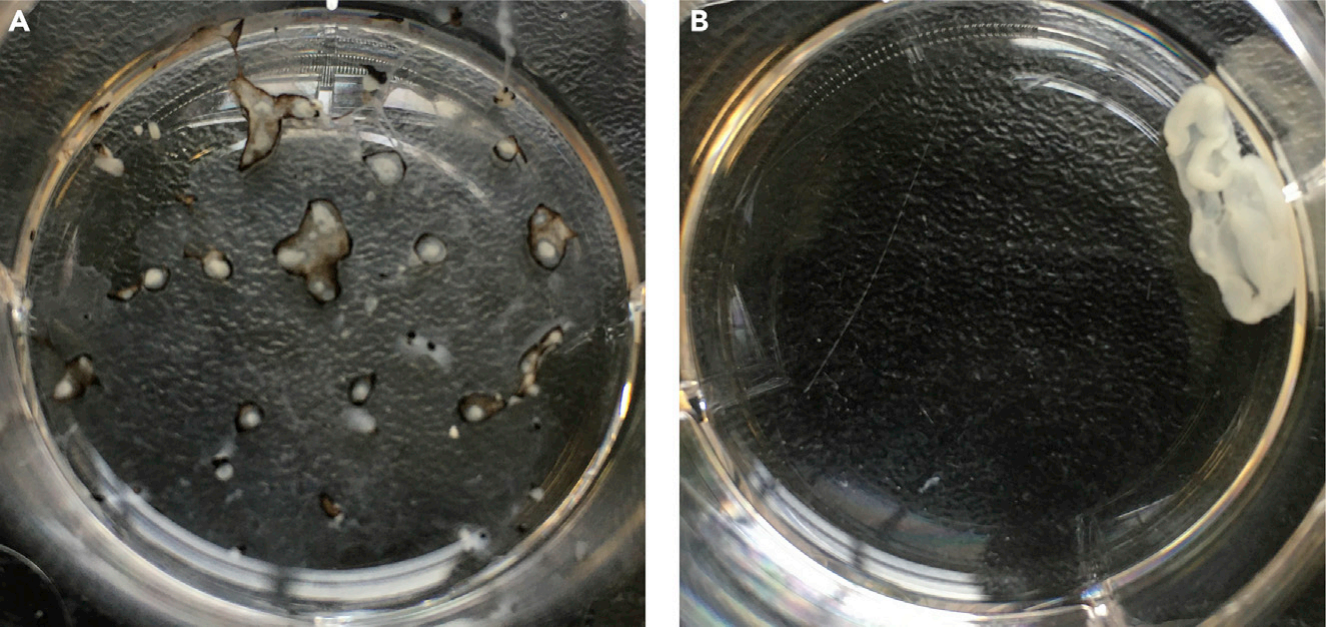
Detaching SEAM cultures (A) SEAM culture that has started to detach from the plate around the edge of the well. (B) Collapsed SEAM culture that has fully detached from the plate.

SEAM cultures can detach from the culture plate when they mature for over 60 days. Detaching can lead to contraction and collapse of the culture due to expression muscle proteins (Figure 9).

### Potential solution

If noticing the culture beginning to detach in the edge of the well, as depicted in Figure 9A, be extra careful when changing the media and moving the plate, to avoid collapse (Figure 9B).

### Problem 2

Isolation and culture of adult human ocular cells, step 12b.

Retina does not separate from RPE-Choroid membrane.

### Potential solution

First soak the eye-cup in calcium and magnesium-free PBS for 5 min. This should loosen the bond between the retina and RPE layer. Next, pinch the RPE/Choroid tissue with a forcep in your non-dominant hand. With either a paint brush or angled forcep in the dominant hand, gently scrape the retina in a downward motion to separate a small area of retina from the RPE/choroid tissue. Once a small area is separated, pinch the retina at that area and gently pull down to enlarge the separation. Rotate the globe and repeat. Eventually the whole retina will be separated and folded inside of the eye cup. This whole procedure must be done with the most delicate touch in order not to tear the tissue.

### Problem 3

Isolation and culture of adult human ocular cells, step 13h.

Primary cultures isolated from donor globes can show very low or no proliferation.

### Potential solution

Proliferation rate can be enhanced by increasing the amount of HI-FBS in TAB-II to 10% and changing the media every day.

### Problem 4

Immunohistochemistry, step 24.

Background in IF images of SEAM cultures is high and compromises image quality.

### Potential solution

The SEAM organoid cultures can grow quite thick, having multiple cell layers on top of each other. If the desired phenotype is present at an earlier developmental time point, fix the culture earlier. In our hands, cultures around 30–35 days old, result in the best image quality in IF without losing cell type diversity.

### Problem 5

Plaque Assay, step 38.

Unexpected cell-type tropism of SARS-CoV-2, attenuated growth in primary cells, changes in viral behavior or larger than normal plaques when performing plaque assays.

### Potential solution

When passaged on Vero E6 cells, SARS-CoV-2 may eliminate a naturally existing furin cleavage site in the spike protein at amino acid positions 681–685 (PRRAR) (Johnson et al., 2021). The same low passage viral stock should be used for all experiments from which the results are to be compared. Before starting experiments, the presence of the furin cleavage site should be confirmed, either by Sanger sequencing of a PCR product that spans the site or by high-throughput sequencing of the entire viral genome.

## Resource availability

### Lead contact

Further information and requests for resources and reagents should be directed to and will be fulfilled by the lead contact, Timothy A. Blenkinsop (timothy.blenkinsop@mssm.edu).

### Materials availability

This study did not generate any new unique reagents.

## Data Availability

The accession number for the single cell RNAseq data sets has been deposited to the National Center for Biotechnology Information (NCBI) under code: GEO: GSE165477.
